# A Zinc-Dependent Metalloproteinase of *Brucella abortus* Is Required in the Intracellular Adaptation of Macrophages

**DOI:** 10.3389/fmicb.2020.01586

**Published:** 2020-07-17

**Authors:** Leonardo A. Gómez, Francisco I. Alvarez, Raúl E. Molina, Rodrigo Soto-Shara, Carla Daza-Castro, Manuel R. Flores, Yrvin León, Angel A. Oñate

**Affiliations:** Laboratory of Molecular Immunology, Department of Microbiology, Faculty of Biological Sciences, University of Concepción, Concepción, Chile

**Keywords:** host-pathogen interaction, virulence, attenuation, toxin-anti-toxin systems, gene regulation

## Abstract

*Brucella abortus* is a pathogen that survives in macrophages. Several virulence factors participate in this process, including the open reading frame (ORF) BAB1_0270 codifying for a zinc-dependent metalloproteinase (ZnMP). Here, its contribution in the intracellular adaptation of *B. abortus* was analyzed by infecting RAW264.7 macrophages with the mutant *B. abortus* Δ270 strain. Results showed that this ZnMP did not participated in either the adherence or the initial intracellular traffic of *B. abortus* in macrophages. Nevertheless, its deletion significantly increased the co-localization of *B. abortus* Δ270 with phagolysosomal cathepsin D and reduced its co-localization with calnexin present in endoplasmic reticulum (RE)-derived vesicles. Although *B. abortus* Δ270 showed an upregulated expression of genes involved in virulence (*vjbR*, *hutC*, *bvrR*, *virB1*), it was insufficient to reach a successful intracellular replication within macrophages. Furthermore, its attenuation favored in macrophages infected the production of high levels of cytokines (TNF-α and IL-6) and co-stimulatory proteins (CD80 and CD86), signals required in T cell activation. Finally, its deletion significantly reduced the ability of *B. abortus* Δ270 to adapt, grow and express several virulence factors under acidic conditions. Based on these results, and considering that this ZnMP has homology with ImmA/IrrE proteases, we discuss its role in the virulence of this pathogen, concluding that ZnMP is required in the intracellular adaptation of *B. abortus* 2308 during the infection of macrophages.

## Introduction

*Brucella abortus* is the causative agent of brucellosis, a highly contagious zoonosis transmitted from bovines to humans. This is a globally distributed disease acquired through the ingestion of contaminated foods or the inhalation of aerosols from secretions of diseased animals (Pappas et al., 2005; Seleem et al., 2010). Infected individuals display irregular fever, headache and arthralgia; its chronicity, associated with the localization of the pathogen in several organs, can produce arthritis, orchitis, endocarditis, hepatitis or encephalomyelitis (Pappas et al., 2005; Franco et al., 2007; de Figueiredo et al., 2015). In animals, it shows a tropism toward the erythritol present in the placenta and genital organs, producing abortions in females and infertility in males (de Figueiredo et al., 2015; Letesson et al., 2017). To reach these organs, *B. abortus* survives within neutrophils, macrophages and dendritic cells, using them as “Trojan horses” to disseminate themselves systemically (Martirosyan et al., 2011; Miraglia et al., 2018; Gutiérrez-Jiménez et al., 2019). Within these cells, endosomal *Brucella* containing vesicles (eBCVs) develop, which interact with early/late endosomes and lysosomes, exposing *B. abortus* to the activity of proteases, reactive oxygen and nitrogen species (ROS and RNS, respectively) and to low pH (von Bargen et al., 2012). In this environment, *B. abortus* expresses virulence factors allowing it to escape from phagolysosomes and reach the endoplasmic reticulum exit sites (ERES), where it develops replicative vacuoles (rBCVs). Additionally, the rBCVs can be engulfed by autophagosome-like structures originating autophagic BCVs (aBCVs), facilitating the exit of *B. abortus* from infected cells and thus completion of its intracellular cycle (von Bargen et al., 2012; Roop et al., 2013; Celli, 2019).

To survive and replicate intracellularly into macrophages, *B. abortus* expresses several virulence factors such as the type IV secretion system (T4SS) virB (Haag et al., 2010; Roop et al., 2013; Celli, 2019). This secretion system is induced by the acidic conditions present in the eBCVs, and regulated transcriptionally, among others, by the vacuolar hijacking *Brucella* regulator (VjbR), *Brucella* luxR-like regulator (BlxR), the two-component system BvrR/BvrS or the histidine utilization regulator (HutC) (Ke et al., 2015). Its expression allows *B. abortus* to translocate several effectors to the cytosol of the host’s cells to control fundamental aspects of the physiology of these cells (von Bargen et al., 2012; Myeni et al., 2013; Ke et al., 2015; Celli, 2019). One of the best studied effectors is BtpA, a protein with Toll/Interleukin-1 receptor (TIR) domain, which is involved in various functions to evade the host’s immune system. This protein interferes with the intracellular signaling depending on Toll-like receptor 2 (TLR2) and TLR4, inactivates the nuclear factor-kappa B (NF-κB), inhibits the secretion of pro-inflammatory cytokines and prevents the maturation of dendritic cells (Cirl et al., 2008; Salcedo et al., 2008, 2013; Sengupta et al., 2013; Alaidarous et al., 2014; Ke et al., 2015). The BtpA protein is codified by the open reading frame (ORF) BAB1_0279 present in genomic island 3 (GI-3) of *B. abortus* 2308. This GI-3 possesses several ORFs, some of which participate in the intracellular survival and replication of *B. abortus* (Salcedo et al., 2008, 2013; Céspedes et al., 2012; Ortiz-Román et al., 2014).

The BAB1_0270 is an ORF codified in GI-3 (*B. abortus* 9-941 BruAb1_0264 and *B. melitensis* 16 M BMEI1683), which plays an important role in the intracellular survival and replication of *B. abortus* 2308 in macrophages and epithelial cells as well as bacterial persistence in the spleen of BALB/c mice two weeks after infection (Ortiz-Román et al., 2014). Although the function of ORF BAB1_0270 in the virulence of this pathogen is unknown, *in silico* analyses show that it codifies a zinc-dependent metalloproteinase (ZnMP) with homology with metalloproteinases of the ImmA/IrrE family present in *Bacillus subtillis* and *Deinococcus* (*D. radiodurans* and *D. deserti*), respectively (Earl et al., 2002; Bose et al., 2008; Ludanyi et al., 2014). These ImmA/IrrE metalloproteases break repressor proteins (ImmR/Ddro, respectively), impeding the horizontal transfer of mobile genetic elements (e.g., ICE*Bs*1) or the transcription of genes required for DNA repair and survival under conditions such as ionizing and ultraviolet radiation or mutations induced by mitomycin C (Earl et al., 2002; Bose et al., 2008; Ludanyi et al., 2014). Hence, if this ZnMP of *B. abortus* 2308 possesses a similar function to ImmA/IrrE, it could participate in the resistance of this pathogen to intracellular microbicide mechanisms of eukaryotic cells, which would explain the attenuation of *B. abortus* Δ270 during the infection of BALB/c mice (Ortiz-Román et al., 2014).

Therefore, in this work we analyze the roles of this ZnMP in the intracellular adaptation of *B. abortus* during the infection of RAW264.7 macrophages with the aim of understanding if it participates in the resistance of *B. abortus* to adverse conditions present in the endosomes/lysosomes. In accordance with the evidence, this ZnMP plays important roles in intracellular survival through increased co-localization of *B. abortus* Δ270 with phagolysosomal cathepsin D and reduced co-localization with calnexin of ER-derived vesicles in addition to the adaptation to an acidic environment. Its attenuation in macrophages significantly favors the expression of the cytokines and co-stimulatory proteins involved in bacterial clearance and required in the activation of T cells.

## Materials and Methods

### Bacterial Strains and Culture Conditions

The strains used in this study were *Brucella abortus* 2308 (wild type, wt), *B. abortus* Δ270 (mutant for ORF BAB1_0270) and *B. abortus* Δ270C (complemented strain) (Ortiz-Román et al., 2014). For some assays, these bacteria were transformed with the broad host range vector pAKgfp1 (Karsi and Lawrence, 2007) (Addgene plasmid #14076), codifying a green fluorescent protein (GFP) which made it possible to study their adherence and intracellular traffic in RAW264.7 macrophages. In addition, the *B. abortus* 2308 strain was inactivated using a 70% methanol-acetone solution (*B. abortus* 2308i) and used as the control in some experiments. All bacteria were cultured in Brucella broth (Difco) and incubated at 37°C, with agitation (120 rpm) for the time needed according to each experiment. *B. abortus* 2308, *B. abortus* Δ270, *B. abortus* Δ270C, and *B. abortus* 2308i will hereafter be called WT, mutant, complemented and inactivated strains, respectively. When necessary, broths were supplemented with antibiotics (50 μg/ml kanamycin and/or 30 μg/ml ampicillin) or their pH was adjusted to 5.5. Characteristics of bacterial strains, plasmids and primers used in this work are described in Table 1. Primers for the mutant strain are described in Supplementary Table S1. All the assays and experiments were done following the procedures established by the Biosafety Committee of the University of Concepción.

**TABLE 1:** Bacterial strains, plasmids and primers used for mutant strains.

Bacterial strains	Characteristics	References
*B. abortus* 2308	Wild type, smooth and virulent strain	Laboratory stock
*B. abortus* Δ270	*B. abortus* 2308 mutant for ORF BAB1_0270 (Km^r^)	Ortiz-Román et al., 2014
*B. abortus* Δ270C	*B. abortus* Δ270 (Km^r^) complemented with pBV1 codifying for ORF BAB1_0270	Ortiz-Román et al., 2014
*B. abortus* 2308-*gfp*	Wild type, smooth and virulent strain transformed with pAKgfp1 plasmid codifying for green fluorescence protein (GFP), (Amp^r^).	This work
*B. abortus* Δ270-*gfp*	*B. abortus* 2308 mutant for ORF BAB1_0270 (Km^r^) and transformed with pAKgfp1 plasmid codifying for green fluorescence protein (GFP) (Amp^r^).	This work
*B. abortus* 2308i	Wild type strain inactivated with 70% methanol-acetone.	This work
*Escherichia coli* BL21 (DE3)	Strain used for recombinant protein production. DE3 is a recombinant phage harboring the T7 RNA polymerase gene.	Studier and Moffatt, 1986

**Plasmids**	**Characteristics**	

pSIM7/pSIM9	Broad-host-range cloning vector (Cm^r^), Lambda Red Recombinase (λ-Red).	Sharan et al., 2009
pKD4	Cassette for Km^r^ sequence	Datsenko and Wanner, 2000
pVB1	Cloning vector for PCR products (Amp^r^)	Ortiz-Román et al., 2014
pVB1-BAB1_0270	Recombinant plasmid (Amp^r^) containing the ORF BAB1_0270.	Ortiz-Román et al., 2014
pAKgfp1	Broad-host-range cloning vector pBBR1MCS4 (Amp^r^) codifying for green fluorescence protein (GFP).	Karsi and Lawrence, 2007
pColdII	Vector derived from backbone plasmid pUC118 is used for cold shock-induced protein expression in *E. coli*followed by a 6xHis tag.	Qing et al., 2004

### Cell Line and Culture Conditions

RAW264.7 murine macrophages (American Type Culture Collection, ATCC) were seeded in 12 or 24-well Nunclon Delta Surface plates (Thermo Fisher Scientific, MA, United States) containing Dulbecco’s Modified Eagle Medium (DMEM) supplemented with 10% fetal bovine serum (FBS) plus 100 UI penicillin, 100 μg/ml streptomycin and 0.25 μg/ml amphotericin B (antibiotic-antimycotic solution, Thermo Fisher Scientific). Cells were incubated at 37°C in a 5% CO_2_ environment for the necessary time in accordance with the experiments described below.

### *In silico* Analysis of the Structure of ZnMP

The physicochemical parameters of this ZnMP, such as amino acid composition, molecular weight (MW), theoretical isoelectric point (pI), extinction coefficient, instability index, *in vitro* and *in vivo* half-life, grand average of hydropathicity (GRAVY) and aliphatic index were evaluated with the ProtParam server at http://web.expasy.org/protparam (Gasteiger et al., 2005). Then, a homology modeling of the hypothetical protein was done using I-TASSER (Iterative threading assembly refinement) server http://zhanglab.ccmb.med.umich.edu/I-TASSER/(Yang et al., 2014), which employs a hierarchical approach to predict the protein structure based on multiple-threading alignments and fragment assembly simulations methods. I-TASSER expresses the confidence of the modeling as a C-score that varies from -5 to 2, where the high C-score is related to high confidence. The visualization of the 3D structures was done with PyMOL. In order to refine the whole hypothetical protein, the best model structure obtained from I-TASSER was introduced into the GalaxyRefine server at http:// galaxy.seoklab.org/cgi-bin/submit.cgi?type=REFINE. This server uses mild and aggressive relaxation methods by reconstruction of side chains (Heo et al., 2013). Later, the final refinement of the model was used for energy minimization with KoBaMIN server at http://chopra-modules.science. purdue.edu/modules/kobamin/html/ (Rodrigues et al., 2012). In order to recognize the potential errors in the initial and final tridimensional structure of the hypothetical protein, the PDB formats were charged in ProSA-web server at https://prosa.services.came.sbg.ac.at/prosa.php (Wiederstein and Sippl, 2007). ProSA-web is based on energy distribution and comparison with the native similar structure of a database. The quality of the 3D predicted protein is shown as a Z-score that indicates possible errors when compared to native experimentally determined proteins stored in a database.

### Purification and Characterization of the ZnMP

The sequence of ORF BAB1_0270 of *B. abortus* (GenBankAM040264.1) was cloned in the vector of expression pColdII (pColdII-ZnMP). This recombinant vector was used to transform the *Escherichia coli* BL21(DE3) strain, where the recombinant protein was purified. Visualization of this recombinant protein was analyzed by Western blot, using anti-6xHis Tag antibodies (Abcam, Cambridge, United Kingdom). Subcellular location, promoter and conserved domains, were analyzed using bioinformatic predictions by CELLO v2.5 (Yu et al., 2004)^1^, BPROM-prediction for bacterial promoters (Solovyev and Salamov, 2011), and the protein-protein Basic Local Alignment Search Tool (BLASTp), respectively. Given that these metalloproteinases contain COG2856 domains, which are usually associated with putative operons with proteins containing Helix-turn-Helix (HTH) domains of the Xre family, a search was conducted to identify this transcriptional regulator in *B. abortus* 2308 and determine if both proteins are part of an operon. These analyses were confirmed by extracting genomic DNA (gDNA) using a Wizard^®^ Genomic DNA Purification Kit (Promega, WI, United States), and total RNA was extracted with TRIzol (Thermo Fisher Scientific Inc., MA, United States) as indicated by the manufacturer. cDNA was obtained from total RNA by RT-PCR using the Maxima First Strand cDNA Synthesis Kit (Thermo Fisher Scientific Inc., MA, United States) and the expression of the BAB1_0270-transcriptional regulator operon was analyzed by PCR amplification of both genes from genomic DNA (gDNA) and cDNA using specific primers (Table 2). PCR products were visualized by agarose gel electrophoresis at 1%.

**TABLE 2:** Primers used in this study for operon identification.

Name of primers	Sequence	Size (bp)
BAB1_0270 F	ATGAGCAGTCAGAATTACGT	549
BAB1_0270 R	TCAGATCCCTTTTTTATTGA	
Regulator F	ATGACCACGGAACTCGGGAA	357
Regulator R	TCATTCCTCTTTACCTCGCC	
Operon F	ATGACCACGGAACTCGGGAA	906
Operon R	TCAGATCCCTTTTTTATTGA	

### Adhesion Assays

The role of ORF BAB1_0270 in the adherence of *B. abortus* strains to RAW264.7 macrophages was examined using strains *B. abortus* 2308-GFP, *B. abortus* Δ270-GFP and *B. abortus* Δ270C-GFP using a protocol described by Arayan et al. (2015). Bacterial adherence was analyzed by culturing 1 × 10^5^ RAW264.7 macrophages adhered to glass coverslips (Thermo Fisher Scientific Inc., MA, United States) and treated with 0.5 mg/ml cytochalasin D, a phagocytosis inhibitor, to impair bacterial internalization. Then, cells were infected with *Brucella*-GFP strains at a multiplicity of infection (MOI) of 1:10 for 30 min at 37°C in 5% CO_2_. Next, the cells were washed using PBS, fixed with 4% paraformaldehyde (PFA) and permeabilized with cold methanol (-20°C) for 10 s. Actin filaments were labeled with phalloidin Alexa Fluor 633 (Thermo Fisher Scientific Inc., MA, United States) diluted at 1:500 for 1 h at 37°C. Finally, the samples were mounted on slides using DakoCytomation fluorescent mounting medium (Dako North America, Inc., United States). All samples were observed using a Zeiss LSM 700 laser scanning confocal microscope (Zeiss, Oberkochen, Germany). Images were acquired and assembled with the IMARIS software from data obtained from three independent experiments.

### Intracellular Trafficking

The contribution of the ORF BAB1_0270 to intracellular traffic was evaluated by co-localization of *B. abortus* 2308-GFP, *B. abortus* Δ270-GFP and *B. abortus* Δ270C-GFP with early endosome antigen 1 (EEA1, an early endosomal protein), cathepsin D (a phagolysosomal protein) and calnexin (an endoplasmic reticulum protein) using confocal microscopy. For this, macrophages where adhered to coverslips and inoculated with different *B. abortus*-GFP strains at a MOI 1:10 for 5, 10, and 15 min for EEA1, and 1 h and 12 h for cathepsin D and calnexin. Post-infection (pi) macrophages were fixed with 4% PFA, permeabilized with 0.1% Triton X-100 and incubated with goat anti-EEA1 polyclonal (Santa Cruz Biotechnology, Dallas, TX, United States), goat anti-cathepsin D (Abcam, Cambridge, United Kingdom) or rabbit anti-calnexin (Abcam, Cambridge, United Kingdom) antibodies for 3 h in a humidity chamber. All antibodies were diluted in PBS (7.4 pH) supplemented with 0.5% bovine serum albumin (BSA). After incubation, the coverslips were washed using PBS (pH 7.4) and incubated with donkey anti-goat IgG Alexa Fluor 594 (Thermo Fisher Scientific Inc., MA, United States) or donkey anti-rabbit IgG Alexa Fluor 647 as secondary antibody (Abcam, Cambridge, United Kingdom) diluted 1:500. Finally, the samples were mounted on slides using DakoCytomation fluorescent mounting medium (Sigma-Aldrich, St. Louis, MO, United States). All samples were observed using a Zeiss LSM 700 laser scanning confocal microscope (Zeiss, Oberkochen, Germany). Images were acquired and assembled with the ImageJ software from data obtained (percentages of co-localization) from two independent experiments.

### Intracellular Survival

The role of the ORF BAB1_0270 in the intracellular survival of *B. abortus* 2308 was studied by infecting RAW264.7 macrophages with wt, mutant and the complemented strains. For this, 2.5 × 10^5^ RAW264.7 macrophages were seeded per well in 24-well plates (Nunclon Delta, Denmark) containing incomplete DMEM medium and incubated for 2 h at 37°C to enable adherence. At the same time, *B. abortus* strains, cultured for 48 h at 37°C, were harvested in the logarithmic phase by centrifugation (2000 × *g* for 10 min), resuspended in incomplete DMEM medium at 2.5 × 10^6^ colony forming units (CFU)/ml and added to the adhered macrophages at MOI 1:10. Then, plates were centrifuged at 500 × *g* for 10 min to facilitate contact between bacteria and macrophages and incubated for 1 h at 37°C in 5% CO_2_. Next, extracellular bacteria were eliminated by replacing the incomplete medium with DMEM medium supplemented with 10% FCS plus 100 μg/ml gentamycin and 50 μg/ml streptomycin. Infected cells were incubated for 6 h and 24 h at 37°C in 5% CO_2_. The intracellular survival of the *B. abortus* strains was evaluated by collecting the cells in phosphate buffer saline-EDTA (PBS, pH 7.4; EDTA 2 mM) by centrifugation (160 × *g* for 10 min) and lysing them with PBS-Triton X-100 (0.1%). Bacterial counts were determined by plating serial dilutions of bacteria in Brucella agar for 72 h at 37°C. All assays were done in triplicate.

### ELISA Cytokines

The contribution of the ORF BAB1_0270 to the attenuation of the mutant strain in the stimulation of infected macrophages was assessed through the production of the pro-inflammatory cytokines TNF-α and IL-6 by ELISA. For this, 5 × 10^5^ RAW264.7 macrophages/well were seeded in 24-well plates (Nunclon Delta, Denmark) and infected with wt, mutant and inactivated strains at MOI 1:10 under the culture conditions described above. The production of these cytokines was quantified in the supernatants from macrophages infected 6 h and 24 h pi. TNF-α and IL-6 production was evaluated by ELISA using the commercial kits eBioscience Mouse TNF alpha and Mouse IL-6 ELISA Ready-SET-Go! (Fisher Scientific, MA, United States). Final concentrations of cytokines were quantified by standard curves based on the concentration of recombinant mouse TNF-α and IL-6. Results were obtained using a VictorX3 ELISA reader (PerkinElmer, Waltham, MA, United States) at 450 nm. All assays were done in triplicate.

### Flow Cytometry

The contribution of the ORF BAB1_0270 to the attenuation of the mutant strain in the stimulation of infected macrophages was assessed through the expression of co-stimulatory proteins CD80 and CD86 by flow cytometry. For this, 5 × 10^5^ RAW264.7 macrophages/well were seeded in 24-well plates (Nunclon Delta, Denmark) and infected with wt, mutant and inactivated strains at MOI 1:10 under the culture conditions described above. Control groups with non-infected (non-stimulated) and lipopolysaccharide (LPS)-stimulated (5 g/ml) (LPS from *E. coli* O26:B6 strain, Sigma-Aldrich) macrophages were included in the experimental design. At 6 and 24 h pi, macrophages were collected from the wells, washed using PBS-EDTA and incubated with Zombie Violet (BioLegend, CA, United States) for viability for 30 min. Cells were washed using PBS-EDTA-BSA and stained with anti-CD11b (clone M1/70) antibodies conjugated to APC/Cy7, anti-CD80 (clone 16-10A1) conjugated to FITC and anti-CD86 (clone GL-1) conjugated to APC (BioLegend, CA, United States) diluted 1:500. Samples were incubated for 30 min in darkness, washed and fixed with 0.5% paraformaldehyde. For data acquisition, cells were resuspended in PBS and analyzed in a BD LSR Fortessa X-20 (BD Biosciences) flow cytometer. The acquired data were analyzed using FlowJo (BD Biosciences). All assays were done in triplicate.

### RT-qPCR Assays

The relative expression of several genes codifying for T4SS virB, its effector proteins and transcription factors involved in the virulence and intracellular survival of *B. abortus* Δ270 was evaluated in infected macrophages by the 2^−ΔΔ*CT*^ method. For this, 1 × 10^6^ RAW264.7 macrophages/well were infected at MOI 1:10 for 24 h with the strains wt, mutant and inactivated. Then, total RNA was extracted with TRIzol (Thermo Fisher Scientific Inc., MA, United States) as was indicated by the manufacturer. Complementary DNA (cDNA) was obtained from RNA by reverse transcription using the Maxima First Strand cDNA Synthesis kit for RT-PCR (Thermo Fisher Scientific Inc., MA, United States) and the relative expression of the genes of interest (Table 3) was quantified using the Takyon q-PCR kit for SYBR assays by means of the AriaMx Real Time PCR system (Agilent Technologies, CA, United States). *gyrA* and *16s* housekeeping genes were used as reference genes for all assays. All assays were done in triplicate.

**TABLE 3:** Primers used in this study for qPCR assays.

Gene (ORF)		Sequences 5′ to 3′
*gyrA* (BAB1_1121)	Forward	cgcaattctatcggtgtgc
	Reverse	atcgccatcgaaatgacct
*16s*	Forward	agctagttggtggggtaaagg
	Reverse	gctgatcatcctctcagacca
*vjbR* (BAB2_0118)	Forward	ttgcgggttgtacggttt
	Reverse	caaggaattgcgtacggtct
*hutC* (BAB2_0308)	Forward	ctggaaatccacgatatacgc
	Reverse	cggttcatcagttcaaaacg
*bvrR* (BAB1_2092)	Forward	gatgaactcttcggcctcaa
	Reverse	aagacgctgcgaaaaagg
*virB1* (BAB2_0068)	Forward	catccatcatcgcagtcg
	Reverse	gctgctctgtcttcagcctta
*virB5* (BAB2_0064)	Forward	gccttcgtctctaccagca
	Reverse	ctaattcgtgctggcgttc
*btpA* (BAB1_0279)	Forward	aagctcctatcaggctaagcaa
	Reverse	tcctgcgcgaccttttta
*vceA* (BAB1_1652)	Forward	acccaatgcgatgcaaag
	Reverse	cgacaactgtaccaaggcatc
*vceC* (BAB1_1058)	Forward	aaatatggaggagttggacacg
	Reverse	tgaaatatcaagcgagctgagt
BAB1_0273	Forward	caatatatcgaagcgcttattgc
	Reverse	cacctgtccatcttcgagaaa
BAB1_0627	Forward	ggttagtgtcggcctgttg
	Reverse	cagaaggttgggcttctgc

### Growth and Gene Expression of *B. abortus* in Acidic Stress

The contribution of the ORF BAB1_0270 to resistance of *B. abortus* 2308 to acidic stress was evaluated by culturing wt, mutant and the complemented strains in 50 ml of Brucella broth adjusted to pH 5.5. Bacteria were cultured at 37°C with agitation (120 rpm) for 96 h. At times 6, 24, 48, 72 and 96 h, 1 ml aliquots were obtained to count bacteria. Serial dilutions were plated on Brucella agar for 72 h and results were reported as CFU/ml. At the same time, supernatant pH from *B. abortus* strain culture was measured using a digital pHmeter. In addition, the relative expression of the genes involved in the virulence of *B. abortus* cultured for 24 h in medium with pH 5.5 (see primers in Table 3) was evaluated by the 2^−ΔΔ*CT*^ method using the RT-qPCR assays described above. For these assays, mRNA from *B. abortus* 2308 cultured under physiological pH was used for gene expression calibration, and *gyrA* and *16s* housekeeping genes were used as reference genes for all assays. All assays were done in triplicate.

### Statistical Analysis

Data obtained from the adherence and intracellular co-localization experiments were analyzed with a *t*-test. Intracellular survival of bacteria, cytokines, co-stimulatory proteins and bacterial growth under acidic stress conditions were analyzed by means of a two-way analysis of variance (ANOVA). Changes in the pH by *B. abortus* strains were measured using a simple linear regression. The gene expression was analyzed by non-parametric Mann-Whitney *U* test. All analyses were done using the GraphPad Prism 8 software. Results were expressed as the mean ± standard deviation. Values of *P* < 0.05 were considered statistically significant.

## Results

### Physicochemical Parameters, Homology Modeling and Purification of ZnMP

**FIGURE 1:**
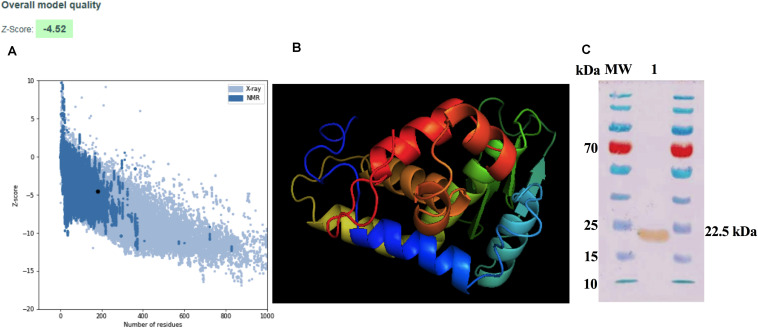
Modeling and purification of Zn-dependent metalloproteinase. **(A)** ProSA-web Z-score plot for 3D structure of the hypothetical protein after refinement. The Z-score of the final model is −4.52. The black dot represents the hypothetical protein. **(B)** 3D structure according to the I-TASSER server after being refined and visualized by Chimera PyMOL. **(C)** Purification of the ZnMP codifying for ORF BAB1_0270 of *B. abortus* 2308. ZnMP sequences were cloned in the expression vector pColdII (pColdII-ZnMP), and the recombinant vector was used to transform the *Escherichia coli* BL21 (DE3) strain. Western blot was performed to visualized protein using anti-6x-HisTag antibodies. MW: molecular weight marker in kDa; 1: recombinant protein purified from *E. coli*.

Physicochemical parameters were evaluated in order to ascertain the conditions for the expression of ZnMP. Results showed that the physicochemical properties of ZnMP have a molecular weight of 21,023.04 Da with an isoelectric point (pI) of 6.01. The half-life of this protein was more than 10 h in *E. coli* (*in vivo*). The aliphatic index and the GRAVY value were 73.96 and -0.423, respectively, indicating the protein stability in a wide range of temperatures and classifies it as hydrophilic. Moreover, the instability index was 37.89, which classifies the hypothetical protein as stable. Prediction of the 3D structure of the hypothetical protein was developed through comparative models with I-TASSER servers. The best model with the highest C-score of 0.00 which was selected for further refinement. The secondary structure of the hypothetical protein contains 7 helixes, 4 strands and 12 coils. The best model predicted by I-TASSER was refinement and energy minimized. The final model was validated with ProSa-web, which showed a Z-score of -4.52 (Figure 1A), placing the hypothetical protein in the same category as native proteins of similar size. The final 3D model is shown in Figure 1B. The purification and Western blot visualization of this ZnMP showed that it has an approximate molecular mass of 22,500 Da (similar to what was predicted by the *in silico* analysis) (Figure 1C) and, based on experimental and Cello v2.5 predictions, is located in the bacterial cytoplasm (4.018 of reliability).

### A ZnMP Forms Part of a Gene Pair With a Xre-Transcriptional Regulator

**FIGURE 2:**
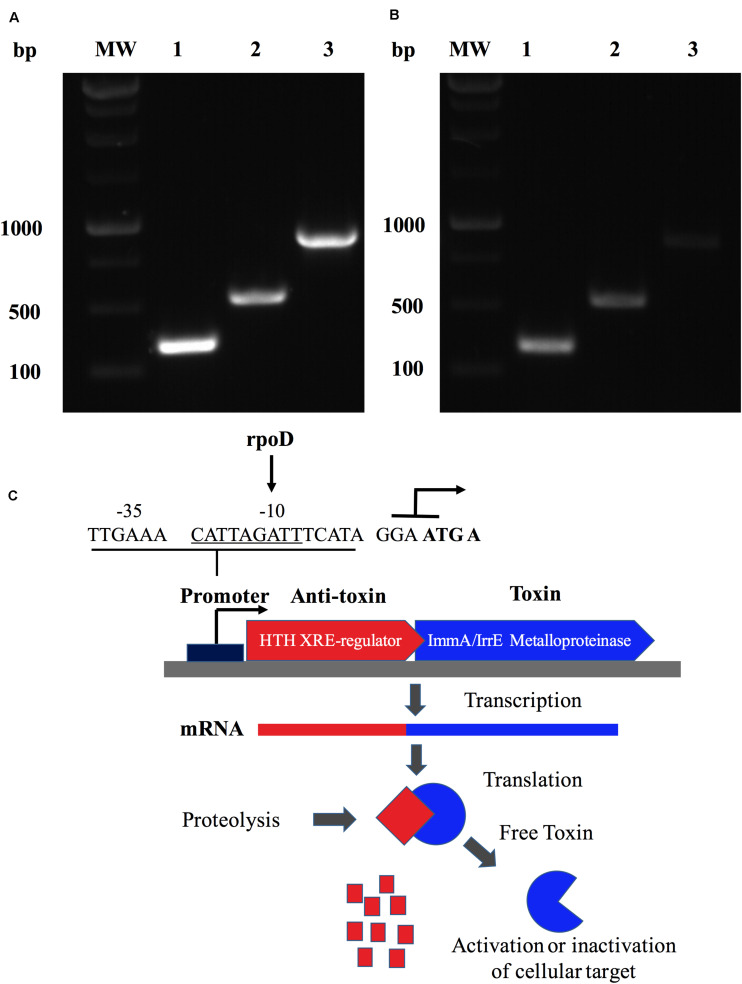
A Zinc-dependent metalloproteinase of *B. abortus* is an operon that forms a putative type II toxin-antitoxin. Identification of the transcriptional unit (operon) constituted by ORF BAB1_0270 and a transcriptional regulator in *B. abortus* 2308 expressed in **(A)** the genomic DNA and **(B)** the cDNA from total RNA. MW: Molecular weight; lane 1: Transcriptional regulator (357 bp); lane 2: BAB1_0270 (549 bp); and lane 3: operon constituted by ORF BAB1_0270-transcriptional regulator (906 bp). **(C)** Hypothetical Type II toxin-antitoxin (TA) model for operon constitute by ZnMP and transcriptional regulator. Toxin (ZnMP) and anti-toxin (transcriptional regulator) are transcribed to mRNA together. Proteases are activated under stress conditions, cleaving the anti-toxin, which increases the levels of toxin-free, inducing various biological functions in bacteria. Predicted promoter at site -35 and -10 binding by RNA polymerase sigma factor rpoD. ATG A: nucleotides shared between final part of transcriptional factor and metalloproteinase codified for ORF BAB1_0270.

The characterization of the ZnMP in a genomic context was studied by *in silico* and experimental methods. Results showed that the ORF BAB1_0270 possesses a 549 bp nucleotide sequence that codifies this ZnMP of 182 amino acids (aa). It has homology with the ImmA/IrrE family of metallopeptidases, which are characterized by the presence of conserved COG2856 domains and HEXXH motifs. Furthermore, a bioinformatics search demonstrated that ZnMP comprises one unit of transcription (operon) with a transcriptional regulator (WP_002967122.1) localized in region 270612-271513 of chromosome I in *B. abortus* 2308 strain (NC_007618.1). These analyses were experimentally confirmed by PCR assays, where the expression of the ORF BAB1_0270 and the transcriptional regulator generated a 906 bp amplicon in the genomic DNA of *B. abortus* 2308 and the same amplicon was obtained from the cDNA (Figure 2A). These results demonstrate that both genes are transcribed together into a single mRNA molecule, where the ORF BAB1_0270 shares four initial nucleotides (ATGA) with the terminal region of the gene codifying for a transcriptional regulator, which is constituted by 357 bp and 118 aa and contains an HTH domain of the Xre family (Figure 2A). Moreover, this operon has a promoter predicted at the -35 and -10 box recognized by RNA polymerase sigma factors rpoD (Figure 2B), and it could be equivalent to the operon codified by the ORFs BruAb1_0264 and BruAb1_0263 of *Brucella abortus* bv. 1 str. 9-941 described by the toxin-antitoxin database as a type II toxin-antitoxin system. A hypothetical Type II toxin-antitoxin (TA) model for this operon constituted by the ZnMP and the transcriptional regulator is described in Figure 2C.

### Adhesion and Intracellular Trafficking of *B. abortus* Δ270 in Macrophages

**FIGURE 3:**
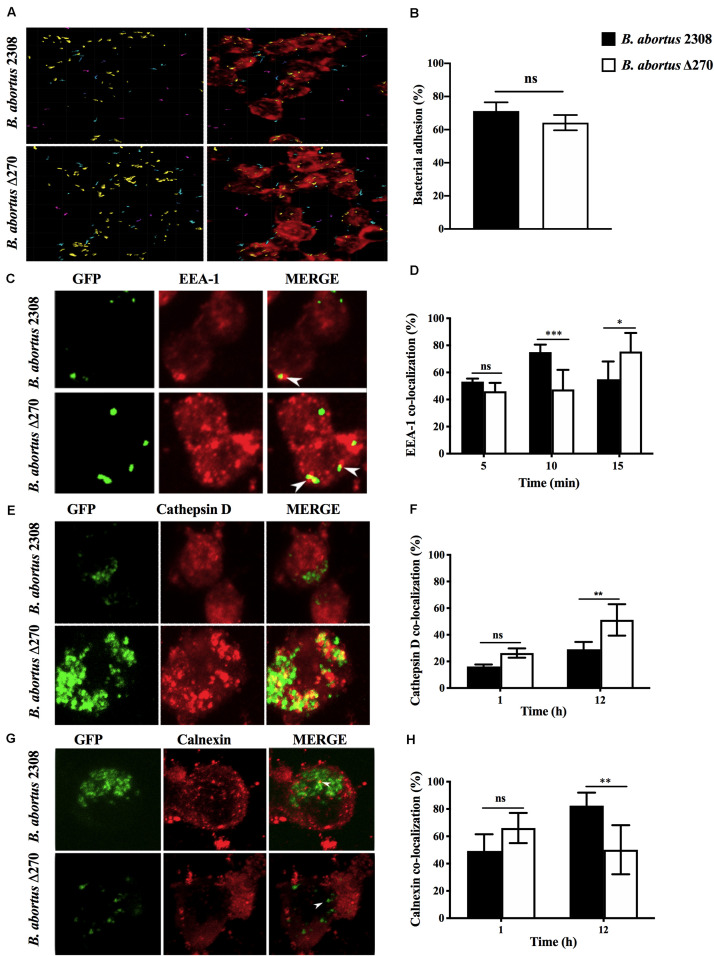
Adhesion and intracellular trafficking of *B. abortus* strains in RAW267.4 macrophages. **(A)** Macrophages were treated with cytochalasin D (0.5 mg/ml) and infected with the *B. abortus* 2308 and *B. abortus* Δ270 strains for 30 min. Image shows the proximity of *B. abortus* strains with the RAW264.7 macrophages, where cyan represents bacteria with a proximity less than 5 mm from macrophages, purple represents bacteria with a proximity greater than 5 mm distance from macrophages, and yellow represents bacteria adhered to macrophages. **(B)** Adherence is expressed by the percentages of bacteria (yellow) adhered to RAW264.7 macrophages. **(C,E,G)** are representative of confocal images showing the co-localization of *B. abortus*-GFP strains with EEA1 at 5, 10, and 15 min pi, cathepsin D at 1 and 12 h pi and calnexin at 1 and 12 h pi. **(D,F,H)** show the percentages of the co-localization of *B. abortus*-GFP strains with EEA1 at 5, 10, and 15 min pi; cathepsin D at 1 and 12 h pi, and calnexin at 1 and 12 h pi. Results are expressed as the mean ± standard deviation. Values of *P* < 0.05 were considered statistically significant, where ns: non-significant differences and *, **, and *** denote values of *P* < 0.05, *P* < 0.01, and *P* < 0.001, respectively. All assays were performed in triplicate.

Cellular infection by *B. abortus* requires adhesion, invasion and the developed of a replicative niche associated with ER-derived vesicles. The role of ORF BAB1_0270 in this process was evaluated by infecting RAW264.7 macrophages with *B. abortus* Δ270. This mutant strain showed levels of adherence to macrophages comparable to the wt strain (*P* > 0.05) (Figures 3A,B). Intracellularly, *B. abortus* Δ270 at 5 min pi showed no significant differences (*P* > 0.05) in levels of co-localization with EEA1 compared to *B. abortus* 2308; however, at 10 min pi it was significantly lower than wt (*P* < 0.001), while at 15 min there were significantly higher differences than wt strain (*P* < 0.05) (Figures 3C,D). Later, at 1 h the mutant and wt strains showed similar levels of co-localization with cathepsin D, a phagolysosomal protease (*P* > 0.05) (Figures 3E,F); however, at 12 h pi differences between the two strains were observed, where the mutant strain showed significantly higher levels of co-localization with cathepsin D than the wt strain (*P* < 0.01) (Figures 3E,F). Additionally, the co-localization of both strains with calnexin, a chaperone protein present in the ER-derived vesicles, was comparable at 1 h pi (*P* > 0.05), but at 12 h pi, the levels of co-localization of the mutant strain were significantly lower than wt (*P* < 0.01) (Figures 3G,H). These results suggest that the deletion of this ZnMP reduces the ability of *B. abortus* Δ270 to escape from phagolysosomal compartments, which would significantly affect its ability to reach the ER-derived vesicles of macrophages.

### Survival and Gene Expression of *B. abortus* Δ270 in Macrophages

**FIGURE 4:**
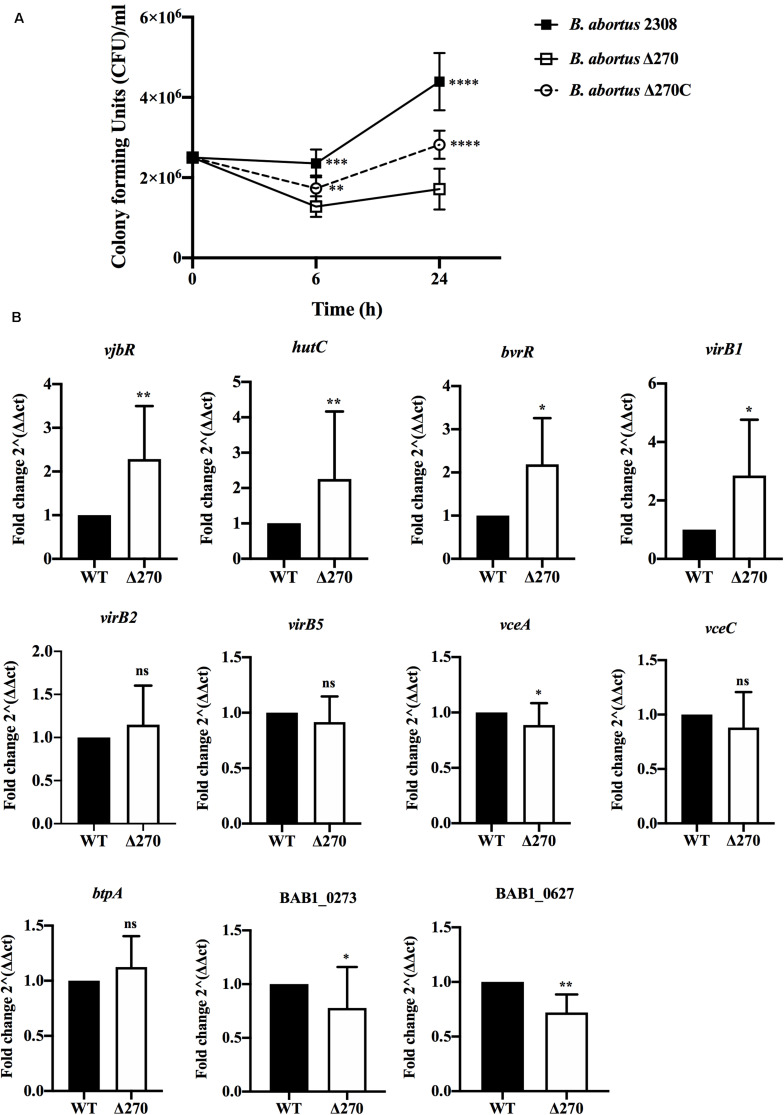
Intracellular survival and gene expression of *B. abortus* strains in macrophages. **(A)** Intracellular bacteria were obtained from macrophages infected at 1:10 MOI with *B. abortus* 2308, *B. abortus* Δ270, or *B. abortus* Δ270C at 6 and 24 h pi. Intracellular survival was recorded as mean ± standard deviation of bacterial counts as CFU/mL. **(B)** The relative expression of *vjbR*, *hutC*, *bvrR*, *virB1*, *virb2*, *virB5*, *vceA, vceC, btpA*, and the ORFs BAB1_0273 (GI-3) and BAB1_0627 induced in *B. abortus* 2308 (wt) and *B. abortus* Δ270 during macrophage infection was calculated by the 2^−ΔΔ*CT*^ method using qPCR assays at 24 h pi. The housekeeping genes *gyrA* and 16s were used as reference genes. Results were expressed as the mean ± standard deviation. Values of *P* < 0.05 were considered statistically significant, where ns denotes non-significant differences, *, **, ***, and **** denote values of *P* < 0.05, *P* < 0.01, *P* < 0.001, and *P* < 0.0001, respectively. All assays were performed in triplicate.

*Brucella abortus* replicates in macrophages through the expression of several virulence factors. The role of the ORF BAB1_0270 in intracellular survival and the expression of several genes involved in virulence was evaluated. At 6 h pi, the intracellular survival of *B. abortus* Δ270 showed CFU/ml counts significantly lower than *B. abortus* 2308 (*P* < 0.001) and the complemented strain (*P* < 0.01). In addition, the *B. abortus* Δ270 counts remained relatively low and constant from 6 h to 24 h, a time where higher differences between *B. abortus* Δ270, the wt and the complemented strains were recorded (*P* < 0.0001) (Figure 4A). At 24 h pi, the *B. abortus* 2308 was characterized by a robust replication, being higher than the mutant and the complemented strains. In this time, the complementary strain showed a higher growth than *B. abortus* Δ270 but lower than *B. abortus* 2308. On the other hand, when the gene expression was measured at 24 h pi, genes *vjbR* (*P* < 0.01), *hutC* (*P* < 0.01) and *bvrR* (*P* < 0.05), genes involved in the response to stress (e.g., acidic stress), quorum sensing (QS) or expression of virulence factors (e.g., T4SS virB) were significantly upregulated in *B. abortus* Δ270 (Figure 4B). Also, the *virB1* gene showed an upregulation in this mutant, but no differences were observed for *virB2* or *virB5* (Figure 4B). Among the genes codifying for effector proteins (*vceA*, *vceC*, and *btpA*), only the *vceA* gene showed a downregulation in *B. abortus* Δ270, whereas genes *vceC* and *btpA* showed no significant differences compared to *B. abortus* 2308 (*P* > 0.05). Finally, the ORF BAB1_0273, codifying for a hypothetical transcriptional regulator, and the ORF BAB1_0627, codifying for a hypothetical protein of unknown functions, were downregulated in *B. abortus* Δ270. These results demonstrate that deletion of ZnMP negatively affects the intracellular survival of *B. abortus* Δ270, which expresses a dysregulation in various genes involved in the infection of macrophages.

### Response of Macrophages Infected With *B. abortus* Δ270

**FIGURE 5:**
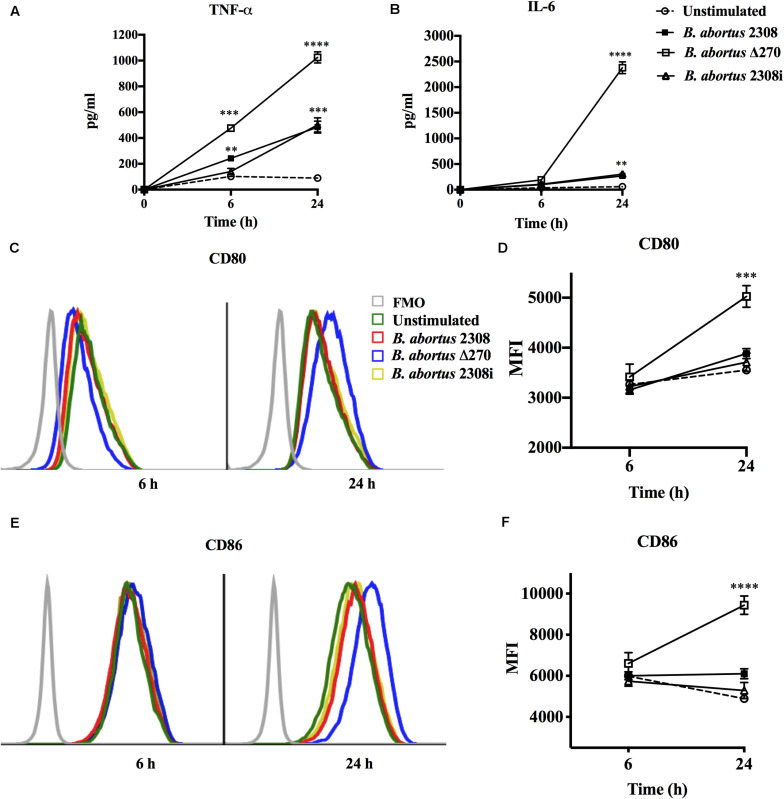
Attenuation of *B. abortus* by deletion of ZnMP in macrophages. Expression of secondary signals for T cells from *B. abortus* strain-infected macrophages at MOI 1:10. **(A)** Production of TNF-α (pg/ml) and **(B)** production of IL-6 (pg/ml) from macrophages infected with *Brucella* strains were measured by Sandwich ELISA at 6 and 24 h pi with standard curves using TNF-α and IL-6 recombinant proteins. **(C,E)** Expression of co-stimulatory proteins CD80 and CD86 from infected macrophages measured by flow cytometry at 6 h pi (blue) and 24 h pi (red). Fluorescence minus one (FMO) was used as a negative control. **(D,F)** show the mean ± standard deviation of the mean fluorescence intensity (MFI) of CD80 and CD86. Data represent unstimulated (PBS-treated) macrophages, *B. abortus* 2308-infected macrophages, *B. abortus* Δ270-infected macrophages and *B. abortus* 2308i (inactivated strain) stimulated macrophages. Results were expressed as mean ± standard deviation. Values of *P* < 0.05 were considered as statistically significant, where **, ***, and **** denote values of *P* < 0.01, *P* < 0.001, and *P* < 0.0001, respectively. All assays were performed in triplicate.

Macrophages destroy bacteria phagocytized in the phagolysosome compartments, which allows them to produce the cytokines and co-stimulatory proteins required to activate T cells. These secondary signals were quantified at 6 and 24 h by ELISA and flow cytometry, respectively. Macrophages infected with the *B. abortus* Δ270 strain at 6 h pi produced significantly higher TNF-α levels than the macrophages infected with wt (*P* < 0.001), inactivated strain (*P* < 0.05) and non-stimulated macrophages (*P* < 0.0001). At 24 h pi, the macrophages infected with the mutant strain secreted even higher levels of TNF-α compared to the different groups (*P* < 0.001) (Figure 5A). Similarly, macrophages infected with the mutant strain at 6 h pi did not secrete significant levels of IL-6 compared to wt or inactivated strains (*P* > 0.05), showing statistically significant differences only with non-infected macrophages (*P* < 0.05). However, at 24 h pi, the macrophages infected with *B. abortus* Δ270 produced highly significant levels of IL-6 compared to the production of this cytokine by macrophages infected with wt, inactivated or non-stimulated macrophages (*P* < 0.0001) (Figure 5B). On the other hand, macrophages infected with *B. abortus* Δ270 at 6 h pi showed no difference in the expression of the CD80 and CD86 proteins compared to macrophages infected with wt, inactivated or non-stimulated macrophages (Figures 5C–F). Moreover, at 24 h pi the expression of CD80 and CD86 in macrophages infected with *B. abortus* Δ270 was significantly higher compared to wt, inactivated or unstimulated macrophages (Figures 5C–F). These results demonstrate that deletion of this ZnMP attenuated the virulence of *B. abortus*, favoring the production of cytokines (IL-6 and TNF-α) and co-stimulatory (CD80 and CD86) proteins, which are secondary signals required in the activation of T cells and bacterial clearance.

### Growth and Gene Expression of *B. abortus* Δ270 Under Acidic Conditions

**FIGURE 6:**
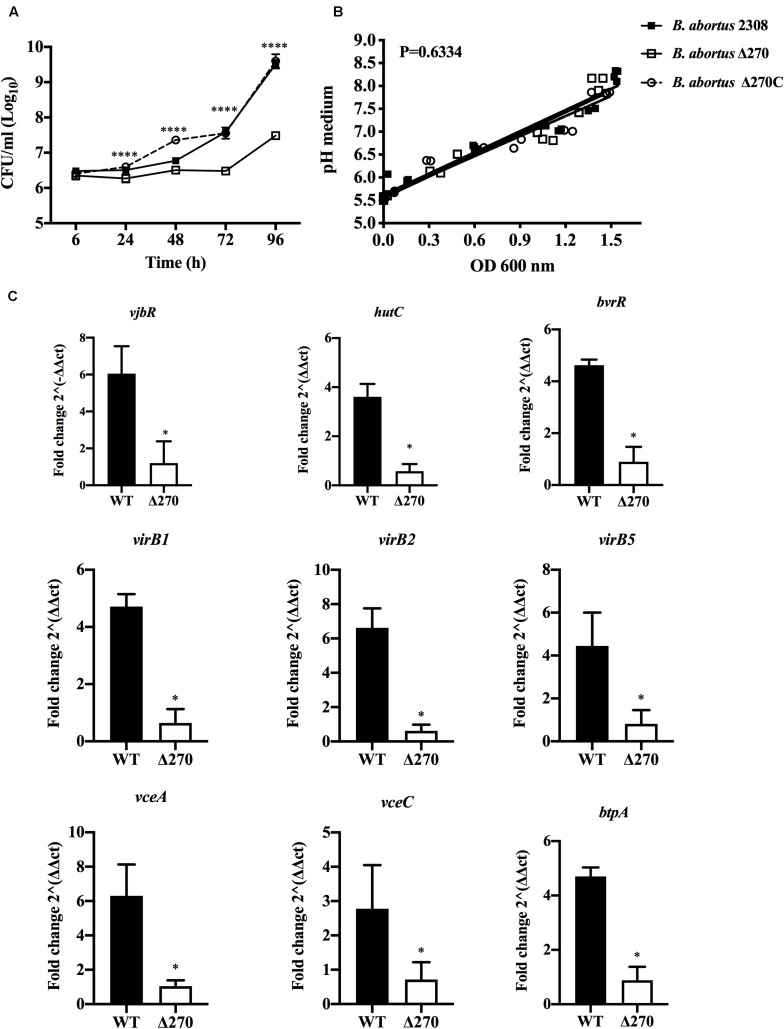
*Brucella* strains growth and gene expression under acidic stress. **(A)** Growth curves of *B. abortus* 2308, *B. abortus* Δ270, and *B. abortus* Δ270C strains cultured in acidic medium (pH 5.5). **(B)** Linear regression analysis of pH changes in the medium during the growth of *Brucella* strains. **(C)** The relative expression of *vjbR*, *hutC*, *bvrR*, *virB1*, *virb2*, *virB5*, *btpA*, *vceA*, and *vceC* induced in *B. abortus* 2308 (wt) and *B. abortus* Δ270 cultured in medium with pH 5.5. The gene expression was calculated by 2^−ΔΔ*CT*^ method using qPCR assays at 24 h. The housekeeping genes *gyrA* and 16s were used as reference genes. Results were expressed as the mean ± standard deviation. *P* value < 0.05 were considered statistically significant, where * denotes values of *P* < 0.05 and **** denotes values of *P* < 0.0001. All assays were performed in triplicate.

*Brucella abortus* is adapted to survive in the acidic conditions present in the endosome/lysosome pathway. Here, the contribution of ORF BAB1_0270 to the growth and gene expression of *B. abortus* Δ270 under acidic conditions was measured. Growth curves of *B. abortus* Δ270 showed a significantly lower ability to adapt and grow in a medium with pH 5.5 compared to wt or the complemented strains (*P* < 0.0001) (Figure 6A). Furthermore, this mutant strain was maintained in the lag phase from 6 to 72 h of culture, entering the logarithmic phase at 72 h (exponential growth). Wt and the complemented strains showed similar growth curves in these acidic pH conditions, with a lag phase that extends to the 48 h time point, and an exponential phase between the 72 and 96 h time points. Interestingly, these strains developed different growth curves in this acidic medium: a linear correlation analysis showed that the three strains increased the pH in the medium, with no significant differences in the pH during the growth of the *Brucella* strains (*P* > 0.05) (Figure 6B). Finally, the relative gene expression of *B. abortus* Δ270 cultured in this medium showed a lower expression of the genes *vjbR*, *hutC*, *bvrR*, *virB1*, *virB2*, *virB5*, *vceA*, *vceC*, and *btpA* than the wt strain (*P* < 0.05) (Figure 6C). These results suggest that deletion of this ZnMP reduces the ability of *B. abortus* to adapt and grow under acidic conditions, an environment that induces in *B. abortus* Δ270 a downregulation of various genes required in virulence and the resistance to acidic conditions.

## Discussion

*Brucella abortus* is a pathogen that has successfully adapted to survive and replicate in the intracellular environment of macrophages (Martirosyan et al., 2011; von Bargen et al., 2012; Roop et al., 2013; de Figueiredo et al., 2015; Celli, 2019). In these cells, this bacterium develops eBCVs, which through their interaction with the endosomes/lysosomes, thereby allowing *B. abortus* to express T4SS virB (Ke et al., 2015). The synthesis of this secretion system is fundamental in the virulence of *B. abortus*, because through it several effector proteins translocate, which plays an important role in intracellular replication and in the control of host cell’s physiology (von Bargen et al., 2012; Myeni et al., 2013; Ke et al., 2015; Celli, 2019). The ORF BAB1_0270 of *B. abortus* 2308, codifying for a ZnMP, participates in the intracellular survival process and in the colonization of the spleen of BALB/c mice (Ortiz-Román et al., 2014). Although the function of this ZnMP in the pathogenicity of *B. abortus* is unknown, its characterization showed that this ZnMP is a cytoplasmic protein with homology with ImmA/IrrE proteases, which modulate the expression of genes involved in resistance to environmental stress (IrrE) (Earl et al., 2002; Bose et al., 2008; Ludanyi et al., 2014). Considering this homology and because *B. abortus* is exposed to harsh conditions (e.g., oxidative and acidic stress) during its intracellular trafficking, in this work we studied if this ZnMP plays a critical role in the intracellular adaptation of this pathogen during the infection process of RAW264.7 macrophages.

In the infection process by *B. abortus* several virulence factors (e.g., Br-LPS or CβG), participate, which initially allow it to adhere and internalize in these cells (Arellano-Reynoso et al., 2005; Lapaque et al., 2005; Haag et al., 2010). In this context, ZnMP does not contribute to either the adherence or the invasion of *B. abortus*, as it requires components from its cellular envelope such as LPS, Hsp60 or SP41 (Castañeda-Roldán et al., 2004; Kim et al., 2004; de Figueiredo et al., 2015). Nevertheless, this ZnMP participates in the intracellular process, being required to escape from the phagolysosomes and survive intracellularly in macrophages. In the phagolysosomes the pathogens are exposed to several factors such as oxidative molecules, pH acid and hydrolytic enzymes, generating a highly microbicide environment for microorganisms (Yates et al., 2005; Huynh and Grinstein, 2007). A key factor in the virulence of *B. abortus* depends on the acidification of eBCVs, which induce the expression of T4SS virB in this bacterium and allow it to escape from the endosome/lysosome pathway and reach the ER-derived vesicles. However, under acidic conditions *B. abortus* Δ270 has shown a reduced ability to grow in culture and a downregulation of genes involved in its virulence in macrophages (e.g., *virB1*, *virB2, virB5*, *vceA, vceV, btpA, vjbR*, *bvrR/bvrS*, or *hutC*). Interestingly, when these genes were evaluated in *B. abortus* Δ270 during the infection of macrophages (at 24 h pi), *vjbR, hutC, bvrR, virB* were upregulated, while BAB1_0273, BAB1_0637 and *vceA* were downregulated. We do not know the function of the ORFs BAB1_0273 or BAB1_0627; however, it has been described that the deletion of the *vceA* in *B. abortus* promotes autophagy and inhibits apoptosis in human trophoblast cells (Zhang et al., 2019). Although *vceA* presented a statistical significance, we do not know if it actually produces a biological change affecting the physiology of *B. abortus*. Therefore, these results indicate that the deletion of ZnMP is required in intracellular trafficking, specifically, to escape from the phagolysosome, inducing in *B. abortus* Δ270 a dysregulation in the gene expression, which negatively affects the expression of several virulence factors involved in the intracellular adaptation of this bacterium in macrophages.

*Brucella abortus* evades the host’s innate immunity by inhibiting the secretion of cytokines IL-1β, IL-6 and TNF-α and co-stimulatory proteins (Barquero-Calvo et al., 2007; Martirosyan and Gorvel, 2013; Ahmed et al., 2016). Nevertheless, *B. abortus* Δ270 is an attenuated strain that showed high levels of co-localization with the phagolysosomes, compartments where these bacteria are degraded and its antigens are recognized by pattern recognition receptors (PRR), activating intracellular signaling pathways such as MAPK (mitogen-activated protein kinase) and MyD88 (Myeloid differentiation primary response 88) and transcription factors such as NF-κB (Macedo et al., 2008; Oliveira et al., 2008; Gomes et al., 2016). The activation of these pathways and factors would trigger the production of IL-6, TNF-α and CD80/CD86 in *B. abortus* Δ270-infected macrophages, whereas *B. abortus* 2308-infected macrophages expressed lower levels of these proteins, demonstrating its ability to evade the host’s immunity. *In vivo*, IL-6, TNF-α, and CD80/CD86 are required in the activation of the interferon gamma-producing T CD4^+^ helper type 1 (Th1) and T CD8^+^ cytotoxic, a protective response induced by the host against brucellosis (Vitry et al., 2012; Skendros and Boura, 2013; Dorneles et al., 2015; Ahmed et al., 2016). Furthermore, IL-6 and TNF-α play several functions in the innate and adaptive immunity, with IL-6 being a cytokine participating in B cell growth, CD4^+^ T cell differentiation and CD8^+^ T cell cytotoxic functions (Dienz and Rincon, 2009; Lee et al., 2016). During infection by *B. abortus*, IL-6 secretion contributes to host resistance, controlling the bactericidal activity of macrophages (Hop et al., 2019). On the other hand, TNF-α is a pleiotropic cytokine that mediates diverse functions in host-pathogen interactions, playing a role as an autocrine and paracrine factor in macrophages (Caldwell et al., 2014). These observations demonstrate that the deletion of this ZnMP significantly reduced the biological fitness of *B. abortus* Δ270 in macrophages, and in turn, through IL-6 and TNF-α expression, could have generated a feedback that activated the infected macrophages, controlling the intracellular growth of *B. abortus* Δ270. Therefore, these results could explain how this attenuated strain during *in vivo* infection is degraded in the phagolysosomes, which activates a protective T cell response which would allow the host to completely remove it from the spleen as described by Ortiz-Román et al. (2014).

The characterization of this ZnMP showed that it forms an operon with a transcriptional regulator containing an HTH domain of the Xre family. Additionally, this operon could constitute a type II toxin-antitoxin (TA II) system, the functions of which in *B. abortus* are unknown. In general, TA II systems participate in several aspects of bacterial physiology, including gene regulation, “growth arrest” and survival under environmental stress, mechanisms contributing to bacterial persistence (Page and Peti, 2016; Harms et al., 2018). These observations open a series of questions about the function of this ZnMP in *B. abortus* during the infection process: (i) Can it play a role similar to the function described for the proteases ImmA/IrrE? If so, (ii) could this ZnMP cleave a gene repressor involved in resistance to environmental stress present in the phagosomes? Regarding this repressor, (iii) could it be the transcriptional Xre-regulator present in the operon, which represses the expression of genes involved in the intracellular adaptation of this bacterium? or, (iv) how does the deletion of ZnMP affect the expression of this Xre-regulator? Finally, if we consider that this ZnMP and the transcriptional Xre-regulator forms a type II TA system, where ZnMP is the toxin and the Xre-regulator is the antitoxin (Figure 2), (v) why does the elimination of ZnMP (toxin) make this mutant strain less virulent and less resistant to intracellular microbicidal mechanisms? Perhaps (vi) this TA II system provides tolerance to stress? Perhaps it plays a role in resistance to environmental stress as occurs with the bacterial SOS response or the production of guanosine pentaphosphate [(p) ppGpp], which contributes to bacterial persistence (Page and Peti, 2016; Harms et al., 2018). Consequently, given the importance of these TA systems, the function of this potential type II TA system present on GI-3 in the physiology of *B. abortus* during its interaction with the host must be clarified.

## Conclusion

In conclusion, this work demonstrates that ZnMP is required in the resistance of *B. abortus* 2308 during its intracellular trafficking in macrophages, as its deletion causes a significantly reduced ability to grow in an acidic medium, escape from the phagolysosomes and replicate intracellularly, a mechanism that attenuated this strain, favoring the expression of proteins required in the bacterial clearance in infected macrophages.

## Author’s Note

This manuscript has been released as a pre-print at BioRxiv (Gómez et al., 2020).

## Data Availability Statement

All datasets presented in this study are included in the article/Supplementary Material.

## Author Contributions

LG and AO contributed to the study conception and design. AO supervised the study. LG, FA, RM, RS-S, CD-C, MF, and YL prepared the material and collected and analyzed the data. LG wrote the first draft of the manuscript. All authors commented on previous versions of the manuscript and read and approved the final manuscript.

## Conflict of Interest

The authors declare that the research was conducted in the absence of any commercial or financial relationships that could be construed as a potential conflict of interest.

## Footnotes

1 http://cello.life.nctu.edu.tw/
