# Supplementary material for: A Zinc-Dependent Metalloproteinase of Brucella abortus Is Required in the Intracellular Adaptation of Macrophages
Source: Front Microbiol. 2020 Jul 17;11:1586. doi: 10.3389/fmicb.2020.01586 (PMC7379133; doi:10.3389/fmicb.2020.01586)
Supplement: Supplementary file 1 [file Data_Sheet_1.PDF]

Supporting information

S1 Table. Primers used for construct of *B. abortus* mutant strain.

Primers name for mutant strains	Sequences	Restriction enzyme	Size (bp)
BAB1_0270 F	GAATTC ATGAGCAGTCAGAATTACGTT	*Eco*RI	561
BAB1_0270 R	GGATCC TCAGATCCCTTTTTTATTGATCC	*Bam*HI	
BAB1_0270 + Km_r F	ATTGGAAAGTCCTCGGCGTT	-----	1659
BAB1_0270 + Km_r R	CGGAAAACGATTCCGAAGCC	-----	
270Kan Forward	CTCAGGCGAGGTAAAGAGGAATGAGCAGTCAGAATTACGTTG TCCCACCGCTTTCGTGGGACAACATCGGTGTAGGCTGGAGCTGCTTC*		1617
270Kan Reverse	GTCTAAGGATTCCACACCGGCAGGTCCCGTTTCAAATCCGATGCA GAAATACGAGGGGTCTGGGCTAGTTCATATGAATATCCTCCTTAG*		

Kanamicyn resistance (Km_r); *Eco*RI and *Bam*HI, restriction endonuclease cleavage sites are underlined.

* Sequence that amplifies the kanamicyn resistance (km_r) cassette are underlined.
